# Mitochondrial dysfunction reduces yeast replicative lifespan by elevating RAS-dependent ROS production by the ER-localized NADPH oxidase Yno1

**DOI:** 10.1371/journal.pone.0198619

**Published:** 2018-06-18

**Authors:** Dae-Gwan Yi, Sujin Hong, Won-Ki Huh

**Affiliations:** 1 Department of Biological Sciences, Seoul National University, Seoul, Republic of Korea; 2 Institute of Microbiology, Seoul National University, Seoul, Republic of Korea; Texas A&M University, UNITED STATES

## Abstract

Mitochondrial dysfunction leads to the accumulation of reactive oxygen species (ROS) which is associated with cellular dysfunction, disease etiology, and senescence. Here, we used the eukaryotic model *Saccharomyces cerevisiae*, commonly studied for cellular aging, to demonstrate how defective mitochondrial function affects yeast replicative lifespan (RLS). We show that RLS of respiratory-deficient cells decreases significantly, indicating that the maintenance of RLS requires active respiration. The shortening of RLS due to mitochondrial dysfunction was not related to the accumulation of extrachromosomal ribosomal DNA circles, a well-known cause of aging in yeast. Instead, intracellular ROS and oxidatively damaged proteins increased in respiratory-deficient mutants. We show that, while the protein kinase A activity is not elevated, ROS generation in respiratory-deficient cells depends on RAS signaling pathway. The ER-localized NADPH oxidase Yno1 also played a role in producing ROS. Our data suggest that a severe defect in mitochondrial respiration accelerates cellular aging by disturbing protein homeostasis in yeast.

## Introduction

Over the past few decades, the budding yeast *Saccharomyces cerevisiae* has contributed to the search for conserved elements in cellular aging [1, 2]. In budding yeast, two distinct lifespan paradigms have been proposed. Chronological lifespan (CLS) is a model for the aging process of post-mitotic cells and measures the amount of time a cell can remain viable in a non-dividing state [3]. Replicative lifespan (RLS) is useful for understanding aging of dividing cells and defined as the number of mitotic divisions that each mother cell can undergo before senescence [4].

In replicative aging, three candidates are considered as important senescence factors. One of them is extrachromosomal ribosomal DNA circles (ERCs) formed by homologous recombination between ribosomal DNA repeats [5]. Based on many studies showing the effects of ERCs level on the replicative age [5, 6], it is believed that the accumulation of ERCs to toxic levels in mother cells leads to senescence. The other candidates of aging factors are oxidatively damaged proteins and protein aggregates [7]. Levels of carbonylated proteins produced by oxidative damage increase with the replicative age of the mother cell [8, 9]. Also, aggregates composed of oxidatively damaged and misfolded proteins are potentially cytotoxic and associated with age-related phenotypes [10]. Given that heavily oxidized proteins tend to form protein aggregates [11], these two aging factors are closely connected with the accumulation of oxidative damage caused by reactive oxygen species (ROS).

A hallmark of aging is the decline in mitochondrial function [12, 13]. One of the well-characterized phenomena associated with mitochondrial dysfunction is the buildup of ROS. Even though some enzymes and processes including membrane-associated NADPH oxidases [14], fatty acid β-oxidation in peroxisomes [15], and the ER protein disulfide resolution system [16] contribute to ROS generation, it has been known that mitochondria are the main cellular source of ROS [17]. Because the accumulation of ROS also leads to the damage to mitochondrial DNA [18], this vicious cycle has been the basis of the mitochondrial free radical theory for several decades [19]. However, a recent research reported that the increase in free radical generation is attributed not to the mitochondrial electron transport chain (ETC) but to the endoplasmic reticulum (ER)-localized NADPH oxidase Yno1 [20], indicating that the issue of ROS accumulation in living cells remains complex and multifactorial.

In this study, we found that severe respiratory disturbance shortens yeast RLS by using several respiratory-deficient mutants. Our results show that the accumulation of ERCs is not the leading cause of reduced RLS in these mutants. On the other hand, respiratory malfunction disrupted the maintenance of RLS by inducing an increase in intracellular ROS and oxidized protein level. Well-known signaling pathways involved in the generation of ROS such as the protein kinase A (PKA) and target of rapamycin (TOR) pathway were not related to ROS accumulation in these mutant cells. Instead, the suppression of RAS signaling reduced ROS production and significantly restored RLS of respiratory-deficient cells. In addition, the majority of detectable ROS was attributed to the ER-localized NADPH oxidase, Yno1. Based on our results, we suggest that the reduced yeast RLS due to mitochondrial dysfunction is caused by the failure to maintain proteostasis.

## Materials and methods

### Yeast strains and growth media

Yeast strains used in this study are listed in S1 Table. Yeast cells were grown in YPD medium (1% yeast extract, 2% peptone, and 2% glucose) or synthetic complete (SC) medium (0.67% yeast nitrogen base without amino acids, 2% glucose, and nutritional supplements) lacking appropriate amino acids for selection [21]. All cultures were incubated at 30°C. Gene disruption was carried out using the one-step PCR-based gene targeting procedure [22]. Strains lacking mitochondrial DNA (rho^0^) were generated by growth in YPD medium supplemented with ethidium bromide (25 μg/ml) [23]. The respiratory deficiency of the strains was confirmed by growth on YPG (1% yeast extract, 2% peptone, and 2% glycerol) medium.

### Cloning and plasmids

Primer sequences used for plasmid construction are shown in S2 Table. The constitutively active Ras2 mutant, Ras2^19V^, was obtained by the QuickChange multisite-directed mutagenesis protocol (Stratagene) and cloned into the *Xba*I and *Sal*I sites of pRS415GPD vector [24]. The overexpression plasmid of C-terminally TAP-tagged SOD1, pRS426ADH-SOD1-TAP, was generated as described previously [25]. pRS423CUP1-6xMYC-cki1^2-200(S125/130A)^ was kindly provided by Dr. Jodi Nunnari [23, 26]. pJU676 (pRS416-SCH9-5HA) has been described previously [27].

### RLS analysis

Analysis of RLS was carried out by micromanipulation as described previously [28], using a Zeiss Tetrad Microscope. All measurements of lifespan were performed on YPD plates. For the effect of respiratory inhibition, antimycin A (3 μg/ml) or oligomycin (10 μg/ml) was added to the plate. Lifespan was determined from five independent experiments (approximately 100 cells per strain in total). Cells that never budded were excluded from the calculation. For statistical analysis, lifespan data sets were compared by one-way ANOVA.

### rDNA silencing assay

Silencing at the rDNA region was tested as described previously [29, 30]. Yeast cells were grown to an OD_600_ of 0.8, and 2.5 μl of 10-fold serial dilutions of the cell suspensions was spotted on the appropriate media. Plates were incubated at 30°C for two days before visualization.

### Quantification of *mURA3* mRNA

Total RNA was extracted from yeast cells using the RNeasy Mini Kit (Qiagen). 1 μg of total RNA was reverse transcribed in a 20 μl reaction mixture containing MLV-reverse transcriptase (M-biotech) and 0.1 μg of oligo-dT (M-biotech) at 42°C for 60 min. The *mURA3* silencing reporter gene harboring the *TRP1* promoter instead of the *URA3* promoter has been described previously [31]. The amount of mRNA was analyzed by quantitative PCR using the Applied Biosystems 7300 Real-Time PCR system (Applied Biosystems). Gene expression was quantified by the 2^-ΔΔC^_T_ method [32] and *ACT1* transcript level was used for normalization of *mURA3* mRNA levels. Primers used for amplification of *mURA3* and *ACT1* are shown in S3 Table.

### rDNA recombination assay

The rDNA recombination rate was determined by measuring the frequency of the loss of *ADE2* integrated at the rDNA locus of strain DMY3010 as described previously [33]. Yeast cells grown to an OD_600_ of ~1.0 in SC medium were spread on SC plates. Colonies were allowed to grow for two days at 30°C and then placed at 4°C for two days to enhance color development. The rDNA recombination rate was calculated by dividing the number of half-red/half-white colonies by the total number of colonies. Entirely red colonies were excluded from all calculations. Three independent experiments were performed, and more than 10,000 colonies were examined for each assay. For statistical analysis, data sets were compared by one-way ANOVA.

### Measurement of intracellular ROS level

Intracellular ROS levels were detected with H_2_DCFDA (2',7'-dichlorodihydrofluorescein diacetate, ThermoFisher Scientific) as described previously [34]. Yeast cells were grown to saturation in YPD medium and diluted to one-hundredth. Then, H_2_DCFDA was added to a final concentration of 10 μg/ml followed by incubation with shaking for one day at 30°C. Intracellular ROS levels were also measured with DHE (dihydroethidium, Sigma-Aldrich) as described previously with some modifications [35]. Yeast cells were grown to saturation and diluted to one-hundredth. Cells were incubated with shaking for 22 h at 30°C, and then DHE was added to a final concentration of 2.5 μg/ml, followed by incubation with shaking for 2 h at 30°C. Fluorescence was analyzed using a BD FACS Canto II flow cytometer (Becton Dickinson). A baseline of zero (background level of fluorescence) was set based on the maximum value of control sample without the ROS indicator. Cells with higher ROS level than background were counted and converted into a percentage.

### Measurement of Sch9 phosphorylation

Analysis of phosphorylated Sch9 was conducted by Western blotting as described previously [27]. Cells were grown to log phase and trichloroacetic acid was added up to 6%. Samples were put on ice for at least 5 min, spun down, washed twice with cold acetone, and dried. Cells were bead-beaten in 100 μl of urea buffer (6 M urea, 50 mM Tris-HCl, pH 7.5, 5 mM EDTA, 1% SDS, 1 mM phenylmethylsulfonyl fluoride, 5 mM NaF, 5 mM NaN_3_, 5 mM *p*-nitrophenyl phosphate, 5 mM Na_2_P_2_O_4_, and 5 mM β-glycerophosphate) followed by heating for 10 min to 65°C. For 2-nitro-5-thiocyanobenzoic acid cleavage, 30 μl of 0.5 M CHES (pH 10.5) and 20 μl of 2-nitro-5-thiocyanobenzoic acid (7.5 mM in H_2_O) were added, and samples were incubated overnight at room temperature before adding 6× sample buffer. Sch9 phosphorylation was detected by SDS-PAGE and immunoblotting using HRP-conjugated mouse anti-HA antibody (sc-7392 HRP, Santa Cruz Biotechnology).

### Western blot analysis and determination of PKA activity

Cell extracts were prepared by suspending cells in lysis buffer (50 mM Tris-HCl, pH 7.5, 150 mM NaCl, 0.01% NP-40, 1 mM EDTA, 1 mM phenylmethylsulfonyl fluoride, 1 mM benzamidine, 1 μg/ml leupeptin, and 1 μg/ml pepstatin), followed by bead-beating. Extracts were spun by centrifugation at 1600 *g* for 10 min at 4°C and the supernatant was subjected to SDS-PAGE. Western blot analysis was performed by standard methods using HRP-conjugated mouse anti-Myc antibody (sc-40 HRP, Santa Cruz Biotechnology) for the detection of Myc-tagged proteins. Images were captured using a luminescent image analyzer, AE-9150 Ez-Capture II (ATTO), and the quantification of phosphorylated protein was performed using CS analyzer 3 software (ATTO).

### Measurement of oxidized proteins

The detection of oxidized proteins (protein carbonyls) was performed using Oxidized Protein Detection Kit (ab178020, Abcam) as described previously [36] with some modifications. Cell extracts were prepared by suspending cells in lysis buffer (50 mM Tris-HCl, pH 7.5, 150 mM NaCl, 0.01% NP-40, 1 mM EDTA, 1 mM phenylmethylsulfonyl fluoride, 1 mM benzamidine, 1 μg/ml leupeptin, and 1 μg/ml pepstatin), followed by bead-beating. Cell lysates were incubated on ice for 20 min and quantified by Bradford assay. About 30 μg/μl of protein was derivatized per sample and mixed with 6× sample buffer. Further procedures for detecting oxidized protein were done by SDS-PAGE and immunoblotting using rabbit anti-DNP antibody and HRP-conjugated goat anti-rabbit antibody (supplied in Oxidized Protein Detection Kit).

## Results

### Mitochondrial respiration is required for the maintenance of RLS

**Fig 1:**
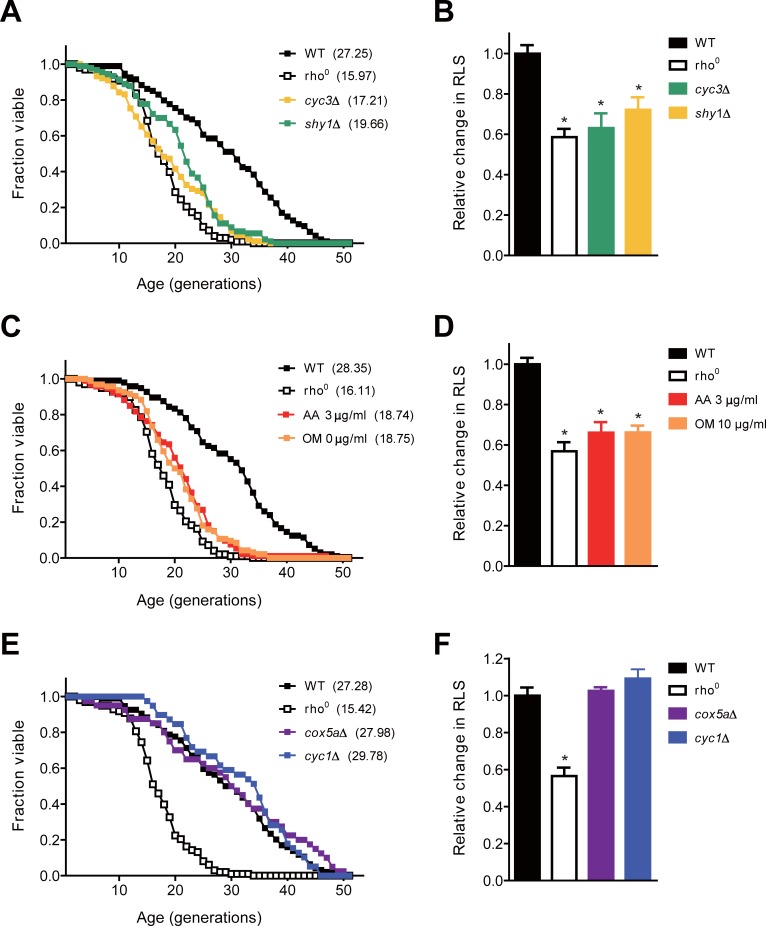
Mitochondrial respiration defect impairs the maintenance of RLS. (A) RLS analysis was performed with wild-type (WT), rho^0^, *cyc3*Δ, and *shy1*Δ cells. (B) The relative changes in RLS were calculated as the ratio of the mean RLS to that of WT cells in (A). (C) RLS analysis was performed with WT, rho^0^, and WT cells on media containing 3 μg/ml antimycin A (AA) or 10 μg/ml oligomycin (OM). (D) The relative changes in RLS of indicated strains were calculated as the ratio of the mean RLS to that of WT cells in (C). (E) RLS analysis was performed with WT, rho^0^, *cox5a*Δ, and *cyc1*Δ cells. (F) The relative changes in RLS of indicated strains were calculated as the ratio of the mean RLS to that of WT cells in (E). Mean RLS values are shown in parentheses. All asterisks indicate *P*<0.01, compared with WT cells (one-way ANOVA).

To confirm whether mitochondrial respiration regulates replicative aging, we first investigated RLS of rho^0^ cells that lack both mitochondrial genome and respiration. Although the loss of mitochondrial DNA is known to impact the longevity of cells in a strain-specific manner [37], RLS of BY4741 rho^0^ cells decreased by about 40% compared to that of wild-type cells (Fig 1A and 1B). Since cells that do not carry cytochrome *c* heme lyase (Cyc3) or cytochrome *c* oxidase (COX) assembly chaperone (Shy1) have been reported to retain no detectable respiration [38], we investigated RLS of *cyc3*Δ or *shy1*Δ mutants in order to exclude the possibility that RLS reduction in rho^0^ cells might be caused by unknown factors other than respiratory malfunction. We confirmed respiratory deficiency of these mutants by the severe growth defect on media containing glycerol, a nonfermentable carbon source (S1 Fig). RLS of each mutant was similar to that of rho^0^ cells (Fig 1A and 1B). Interestingly, RLS of wild-type cells was significantly reduced by the addition of inhibitors such as antimycin A and oligomycin that specifically block mitochondrial respiration (Fig 1C and 1D). These results suggest that cellular respiration is important for the maintenance of RLS in yeast.

We also examined RLS of cells with mutations that are associated with mitochondrial respiration but do not cause severe respiratory failure. These mutants include cells lacking the subunit Va of COX (Cox5a) or iso-1-cytochrome *c* (Cyc1) [38]. Although the viability of *cox5a*Δ or *cyc1*Δ cells is reduced in the medium containing glycerol (S1 Fig), both strains showed no noticeable change in RLS compared to wild-type cells (Fig 1E and 1F). This result suggests that respiratory failure above a certain threshold value is required to induce a reduction in replicative aging.

### rDNA silencing is not a major cause for RLS reduction in respiratory-deficient cells

**Fig 2:**
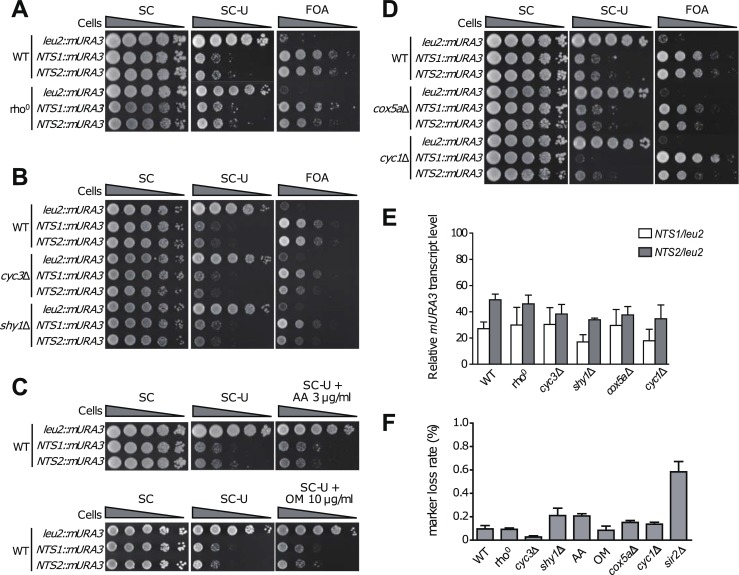
Transcriptional silencing is not a major cause for shortening of RLS in respiratory-deficient cells. Silencing at the rDNA region was assessed by monitoring the growth of 10-fold serial dilution of cells on SC media lacking uracil or supplemented with FOA. SC medium was used as a control. (A) 10-fold serial dilutions of wild-type (WT) and rho^0^ cells were spotted on SC media without uracil or with FOA. (B) 10-fold serial dilutions of WT, *cyc3*Δ, and *shy1*Δ cells were spotted on SC media without uracil or with FOA. (C) 10-fold serial dilutions of WT cells were spotted on SC media without uracil containing 3 μg/ml antimycin A (AA) or 10 μg/ml oligomycin (OM). (D) 10-fold serial dilutions of WT, *cox5a*Δ, and *cyc1*Δ cells were spotted on SC media without uracil or with FOA. (E) Total RNA was extracted from WT, rho^0^, *cyc3*Δ, *shy1*Δ, *cox5a*Δ, *cyc1*Δ, and *sir2*Δ cells. Quantitative real-time reverse transcription-PCR analysis was performed to measure the *mURA3* transcript level. Amplification efficiencies were validated and normalized against *ACT1*. The relative transcript levels of the *mURA3* gene were calculated as the ratio of the normalized transcript levels of the *mURA3* gene inside the rDNA array (*NTS1*::*mURA3* and *NTS2*::*mURA3*) to that outside the rDNA array (*leu2*::*mURA3*). Values represent the average of three independent experiments, and error bars indicate the standard deviation. (F) rDNA recombination assay was performed to check rDNA stability of the indicated cells. rDNA recombination is represented by the frequency of loss of the *ADE2* marker gene integrated at the rDNA locus in the corresponding cells. Values represent the average of three independent experiments, and error bars indicate the standard deviation. Asterisks indicate *P*<0.01, compared with WT cells (one-way ANOVA).

One of the factors that influence yeast longevity is rDNA silencing [5]. To examine whether respiratory deficiency worsens the stability of rDNA, we carried out an rDNA silencing assay using the *mURA3* silencing reporter gene. For this test, we used yeast strains harboring the *mURA3* silencing reporter gene integrated either into the non-transcribed spacer regions (NTS1 and NTS2) of the rDNA locus or outside the rDNA array [29]. *CYC1*, *CYC3*, *COX5A*, or *SHY1* genes were deleted from each strain, and cells of each strain were 10-fold serially diluted and spotted on SC medium in which uracil was omitted or 5-fluoroorotic acid (FOA) was added. In wild-type cells, the *mURA3* reporter gene was effectively silenced at both the NTS1 and NTS2 regions, as indicated by decreased growth on uracil-deficient medium and increased growth on medium containing FOA (Fig 2A). Compared to wild-type cells, rho^0^ cells did not exhibit significant changes in growth on medium lacking uracil or containing FOA (Fig 2A), suggesting that rDNA silencing has a low correlation with RLS reduction due to mitochondrial respiratory failure. Likewise, no significant changes in rDNA silencing were observed in respiratory-deficient *cyc3*Δ and *shy1*Δ cells (Fig 2B) or wild-type cells treated with respiratory inhibitors (Fig 2C). *cox5a*Δ and *cyc1*Δ cells with little respiratory defect also did not show a remarkable difference in rDNA silencing from wild-type cells, except that rDNA silencing was slightly increased at the NTS1 region in *cyc1*Δ cells (Fig 2D). Given that RLS of *cox5a*Δ and *cyc1*Δ cells did not differ significantly from that of wild-type cells (Fig 1E and 1F), these mutations do not seem to be related to rDNA silencing.

To more directly examine rDNA silencing, we measured the transcript levels of the *mURA3* gene by using a real-time reverse transcription-PCR analysis as described previously [39]. In wild-type cells, the transcription of the *mURA3* gene at the NTS1 and NTS2 regions was effectively silenced (>50%) compared to that outside rDNA (Fig 2E). The relative transcript levels of *mURA3* were not significantly changed in mitochondrial respiratory-deficient rho^0^, *cyc3*Δ, and *shy1*Δ cells showing reduced RLS, compared to that of wild-type cells. The transcription of *mURA3* was also not significantly changed in *cox5a*Δ and *cyc1*Δ cells that do not show RLS reduction.

To further test rDNA stability in respiratory-deficient cells, the frequency of loss of the *ADE2* marker gene integrated at the rDNA locus was monitored. As reported previously [29, 40], mutant cells without Sir2, a major rDNA silencing factor, exhibited a considerable increase in the frequency of *ADE2* marker loss compared to wild-type cells (Fig 2F). However, in agreement with the above observations that rDNA silencing is unrelated to mitochondrial respiratory defect, the frequency of *ADE2* marker loss in respiratory-deficient cells was not significantly different from that of wild-type cells. Taken together, these results suggest that rDNA silencing, a well-known longevity factor in yeast, is not related to RLS reduction caused by mitochondrial respiratory failure.

### Mitochondrial respiratory deficiency induces an increase in the amount of intracellular ROS regardless of TOR and PKA pathways

**Fig 3:**
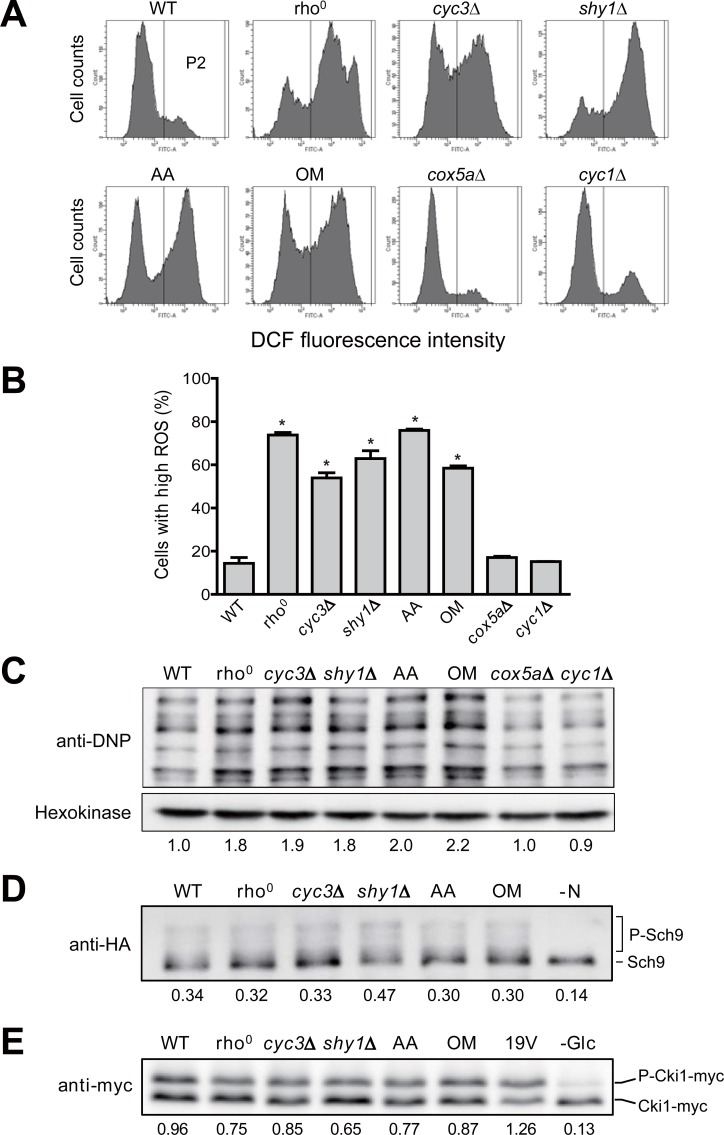
Increased ROS level decreases RLS in respiratory-deficient cells. (A) Intracellular ROS levels in wild-type (WT), rho^0^, *cyc3*Δ, *shy1*Δ, *cox5a*Δ, *cyc1*Δ, and WT cells treated with 3 μg/ml antimycin A (AA) or 10 μg/ml oligomycin (OM) were detected with H_2_DCFDA. Fluorescence was analyzed using a BD FACS Canto II flow cytometer. (B) Cells with high ROS were calculated as a percentage of cells with higher fluorescence intensity than the maximum fluorescence intensity of control sample without the ROS indicator. Values represent the average of three independent experiments, and error bars indicate the standard deviation. All asterisks indicate *P*<0.01, compared with WT cells (one-way ANOVA). (C) Carbonylated proteins in WT, rho^0^, *cyc3*Δ, *shy1*Δ, *cox5a*Δ, *cyc1*Δ, and WT cells treated with 3 μg/ml AA or 10 μg/ml OM were detected using Oxidized Protein Detection kit. Hexokinase was used as a loading control. The relative ratio of carbonylated proteins in the indicated strain to those of WT cells is shown below each lane. Data are representative of at least three independent experiments. (D) Total protein was extracted from WT, rho^0^, *cyc3*Δ, *shy1*Δ, and WT cells treated with 3 μg/ml AA or 10 μg/ml OM, and WT cells under nitrogen starvation. All cells harbor pRS416-SCH9^T570A^-5HA. Immunoblotting was performed using a mouse anti-HA antibody. Data are representative of at least three independent experiments. (E) Total protein was extracted from WT, rho^0^, *cyc3*Δ, *shy1*Δ, WT cells treated with 3 μg/ml AA or 10 μg/ml OM, WT cells expressing constitutively active *RAS2*^*val19*^ (19V), and WT cells under glucose starvation. All cells harbor pRS423-CUP1-6xMYC-cki1^2-200(S125/130A)^. Immunoblotting was performed using a mouse anti-Myc antibody. The relative ratio of phosphorylated to unphosphorylated forms of Cki1 is shown below each lane. Data are representative of at least three independent experiments.

Given that the accumulation of ROS contributes to the buildup of other aging factors such as oxidatively damaged proteins and protein aggregates [7], we examined whether defective respiration induces intracellular ROS production. To detect ROS accumulation, we employed flow cytometry using H_2_DCFDA, a fluorescent probe that reacts with several ROS such as hydroxyl radicals and H_2_O_2_ [41]. Cell population (P2) showing higher fluorescence than background level was converted into a percentage (S2A Fig). In wild-type cells, about 14% of cell population emitted higher fluorescence than background level (Fig 3A and 3B). Remarkably, respiratory-deficient rho^0^, *cyc3*Δ, and *shy1*Δ cells showed 4~5 fold increase in P2 percentage. It was also found that the level of intracellular ROS increased significantly when wild-type cells were treated with antimycin A or oligomycin. In contrast, *cox5a*Δ and *cyc1*Δ cells, which showed no decrease in RLS, did not exhibit a significant change in P2 percentage. In addition, we employed another fluorescent probe DHE to detect intracellular superoxide radical. Similar to the above results with H_2_DCFDA, cells with a severe defect in respiration showed increased percentages of the high-ROS cell population, whereas a significant change was not observed in *cox5a*Δ and *cyc1*Δ cells (S2B–S2D Fig). These observations suggest that the reduction of RLS in respiratory-deficient mutants may rely on the elevated level of intracellular ROS.

To test a possibility that elevated ROS production might increase the level of oxidatively damaged proteins in cells with respiratory failure, we analyzed protein oxidation by using the oxyblot assay. When proteins are oxidized by ROS, carbonyl groups such as aldehydes and ketones are produced on protein side chains [42]. These groups can be derivatized with 2,4-dinitrophenylhydrazine to form a stable 2,4-dinitrophenyl (DNP) hydrazone product and be measured with the anti-DNP antibody by western blot immunoassay. Consistent with the above results that a severe defect in mitochondrial respiration induces ROS accumulation, the levels of protein oxidation in respiratory-deficient rho^0^, *cyc3*Δ, and *shy1*Δ cells were about two times higher than that in wild-type cells (Fig 3C and S3A Fig). Treatment of antimycin A and oligomycin mimicking respiratory failure also increased intracellular protein oxidation. In case of *cox5a*Δ and *cyc1*Δ cells, however, the oxidized protein level was not significantly different from that of wild-type cells. These results support our hypothesis that increased ROS by mitochondrial respiratory deficiency induces protein oxidation and thus affects RLS.

It is known that the activation of TOR and PKA pathways leads to the accumulation of ROS in yeast [43, 44]. We examined whether these two pathways are involved in the elevation of ROS level due to respiratory defect. Given that Sch9 is a major substrate of TOR kinase, Sch9 phosphorylation was quantitatively analyzed using SDS-PAGE mobility shift assay in order to measure the activity of TOR kinase [27]. As expected, the up-shifted, phosphorylated forms of Sch9 were observed in wild-type cells (Fig 3D and S3B Fig). Consistent with previous reports that nitrogen starvation inhibits the activity of TOR kinase [45], the level of phosphorylated Sch9 was reduced under nitrogen starvation. Notably, a significant change in Sch9 phosphorylation was not observed in respiratory-deficient rho^0^, *cyc3*Δ, and *shy1*Δ cells or in cells treated with respiratory inhibitors. This observation suggests that TOR pathway is not involved in the elevation of ROS level caused by respiratory failure.

For the determination of PKA activity, we used a PKA substrate reporter derived from a native substrate Cki1 [26]. By analyzing the mobility shift on SDS-PAGE, PKA-dependent phosphorylation of the Cki1 reporter was detected and the ratio of phosphorylated and unphosphorylated forms was calculated. As expected, cells with a constitutively active variant of Ras2^19V^ [46, 47] exhibited a considerable increase in the phosphorylated form of Cki1, while a nearly 90% reduction in the phosphorylated form of Cki1 was observed in cells under glucose starvation (Fig 3E and S3C Fig). However, we could not observe a significant change in Cki1 phosphorylation not only in respiratory-deficient rho^0^, *cyc3*Δ, and *shy1*Δ cells but also in cells treated with respiratory inhibitors. This result suggests that, like TOR pathway, PKA pathway is not related to ROS accumulation induced by mitochondrial respiratory deficiency.

### RAS signaling and NADPH oxidase Yno1 contribute to intracellular ROS accumulation

**Fig 4:**
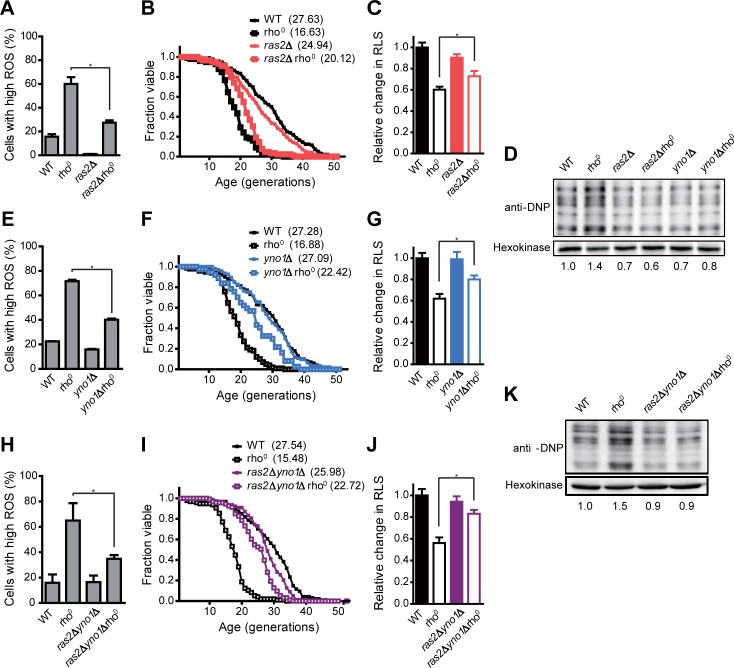
RAS signaling pathway and Yno1 contribute to the buildup of intracellular ROS in cells lacking mitochondrial respiration. (A, E, and H) Intracellular ROS levels in the indicated strains were detected with H_2_DCFDA. Fluorescence was analyzed using a BD FACS Canto II flow cytometer. Cells with high ROS were calculated as a percentage of cells with higher fluorescence intensity than the maximum fluorescence intensity of control sample without the ROS indicator. Values represent the average of three independent experiments, and error bars indicate the standard deviation. All asterisks indicate *P*<0.01, compared with rho^0^ cells (one-way ANOVA). (B, F, and I) RLS analysis was performed with the indicated strains. (C, G, and J) The relative changes in RLS were calculated as the ratio of the mean RLS to that of WT cells. Mean RLS values are shown in parentheses. All asterisks indicate *P*<0.01, compared with rho^0^ cells (one-way ANOVA). (D and K) Carbonylated proteins in indicated strains were detected using Oxidized Protein Detection kit. Hexokinase was used as a loading control. The relative ratio of carbonylated proteins in the indicated strain to those of WT cells is shown below each lane. Data are representative of at least three independent experiments.

Above, we have shown that PKA activity does not contribute to the elevation of ROS level in respiratory-deficient cells. It is well established that Ras2 activates adenylyl cyclase depending on the levels of glucose and regulates the activation of PKA by controlling cAMP levels in yeast [48]. However, a recent study reported that RAS signaling operates independently of PKA to promote ROS accumulation in cells lacking COX activity [20]. To test whether RAS signaling is responsible for ROS accumulation in respiratory-deficient cells, we measured intracellular ROS level in *RAS2*-deleted rho^0^ cells. Interestingly, the loss of Ras2 resulted in a significant reduction of ROS level in rho^0^ cells (Fig 4A, S4A and S5A and S5B Figs). *RAS2* deletion also led to a significant recovery in RLS of rho^0^ cells (Fig 4B and 4C). Consistent with our notion that ROS accumulation impairs the maintenance of RLS by promoting protein oxidation, the level of oxidized proteins was lowered by about 60% in *RAS2*-deleted rho^0^ cells compared to rho^0^ cells (Fig 4D and S6A Fig). These results suggest that RAS signaling contributes to ROS accumulation and concomitant protein oxidation induced by mitochondrial dysfunction.

Intriguingly, although *ras2*Δ cells have quite low ROS level compared to wild-type cells, RLS of *ras2*Δ cells was not increased but even slightly decreased (Fig 4B and 4C). Ras2 has been known not only as a key component of generating ROS in respiratory-deficient cells but also as a major regulator responsive to external environment [48]. One of the downstream effectors of Ras2 is PKA which plays crucial roles in a wide variety of cellular processes [49]. Moreover, there is a complex network that covers multiple regulatory pathways in the downstream of Ras2 [50]. Therefore, even though *ras2*Δ cells have lower ROS level than wild-type cells, *ras2*Δ cells seem to exhibit reduced RLS compared to wild-type cells because of defects in several regulatory pathways important for normal cell function. It is presumable that the effect of defective cell function overrides the beneficial effect of reduced ROS in *ras2*Δ cells.

RAS signaling has been involved in the regulation of the endoplasmic reticulum-associated degradation (ERAD) pathway which plays an important role in ER quality control mechanisms [51]. According to a previous study, among other ER-associated oxidases under the control of the ERAD pathway, ER-localized NADPH oxidase Yno1 is required for ROS generation in COX-deficient cells [20]. Based on these reports, we next examined whether Yno1 plays a role in the buildup of ROS in cells with respiratory malfunction. Notably, *YNO1* deletion led to about 50% decrease in ROS accumulation in rho^0^ cells (Fig 4E, S4B, S5A and S5B Figs). RLS of *yno1*Δ rho^0^ cells was also significantly restored compared to that of rho^0^ cells (Fig 4F and 4G). Furthermore, we observed a considerable reduction in the level of protein oxidization in *YNO1*-deleted rho^0^ cells compared to rho^0^ cells (Fig 4D and S6A Fig), suggesting that Yno1 is involved in ROS accumulation and protein oxidation in respiratory-deficient cells.

Next, we checked whether RAS signaling and Yno1 contribute independently to ROS accumulation and concomitant RLS reduction in rho^0^ cells. To test this, we measured intracellular ROS level and RLS of *ras2*Δ *yno1*Δ rho^0^ cells. With respect to ROS reduction, no synergistic effect was observed in *ras2*Δ *yno1*Δ rho^0^ cells compared to *ras2*Δ rho^0^ or *yno1*Δ rho^0^ cells (Fig 4H, S4C, S5A and S5B Figs). In addition, we could not observe the synergistic effect of deletion of *RAS2* and *YNO1* on RLS of rho^0^ cells (Fig 4I and 4J). In accordance with the above observations, the synergistic effect of deletion of *RAS2* and *YNO1* on protein oxidation of rho^0^ cells was not detected (Fig 4K and S6B Fig). These results suggest that RAS signaling and Yno1 act on the same pathway in the buildup of ROS and consequent reduction of RLS induced by mitochondrial dysfunction. These results are also consistent with a recent report that RAS signaling is associated with degradation of Yno1 through the ERAD pathway [20].

## Discussion

Although not yet clearly identified, mitochondria are believed to play important roles in cellular aging. In this study, we used the budding yeast *S*. *cerevisiae* to investigate the effects of mitochondrial respiratory deficiency on replicative aging. Through analysis of RLS in respiratory-deficient mutants, we found that mitochondrial respiration is required for the maintenance of RLS (Fig 1). Our data also show that rDNA silencing, one of the major aging factors, is not relevant to RLS reduction induced by defective mitochondria (Fig 2). Instead, we suggest that the elevated ROS levels triggered by mitochondrial malfunction are responsible for the reduction of RLS by impairing proteostasis (Fig 3A, 3B and 3C). Given that mitochondrial respiration is also essential to sustain yeast CLS [35, 52], our results highlight that the state of mitochondrial respiration is widely involved in the longevity process.

In contrast to our results, some studies reported that RLS of mitochondrial respiratory-deficient cells is maintained or even increased depending on laboratory strains used [37, 53, 54]. Nonetheless, the accumulation of ROS is observed in respiratory-deficient cells derived from several laboratory strains and this phenomenon is also conserved in higher eukaryotes [20, 35, 55]. Previous studies have found that cells lacking mitochondrial respiration generally have low viability due to hypersensitivity to various stresses such as hydrogen peroxide, ethanol, and heat [38, 56, 57]. Furthermore, the tolerance for intracellular ROS varies depending on the difference of genetic background among the laboratory strains [58]. Taking all of the above into consideration, it is plausible that RLS of respiratory-deficient mutants is influenced by the resistance to accumulated ROS, which is different among the laboratory strains. Meanwhile, it has been reported that a mild inhibition of mitochondrial respiration prolongs the lifespan of several organisms [37, 59–62]. Consistent with these reports, although *cox5a*Δ and *cyc1*Δ cells had some defects in respiration (S1 Fig), these cells did not exhibit RLS reduction nor ROS accumulation (Figs 1C and 3). In addition, thresholds of mitochondrial respiration are necessary to regulate yeast CLS [38]. Therefore, the degree of respiratory capacity seems to be another determining factor for lifespan regulation in cells with respiratory defects.

We observed that mitochondrial dysfunction has no significant effect on the activation of TOR and PKA kinases (Fig 3D and 3E). On the basis of these results, we suggest that the buildup of ROS in respiratory-deficient cells is not attributed to TOR and PKA pathways. Instead, it is plausible that the RAS signaling pathway regulates the activity of NADPH oxidase Yno1 responsible for ROS overproduction in respiratory-deficient cells (Fig 4). Although Yno1 is known to be regulated by ERAD-mediated degradation under the control of RAS signaling [20, 51], the underlying mechanism is expected to be more complicated. It has been reported that the activity of RAS is involved in actin dynamics and remodeling upon nutrient depletion [63, 64]. In addition, previous genome-wide screening studies reported that *yno1*Δ cells show a hypersensitivity to inhibitors of the actin cytoskeleton and that Yno1 functionally regulates the nucleation and elongation step in the biosynthesis of F-actin cables [65, 66]. Taken together, these findings suggest that the interactive regulation between RAS signaling and Yno1 can be linked through actin dynamics. Meanwhile, the mitochondrial retrograde pathway is known to regulate many cellular activities and aging in yeast [67]. Given that the retrograde response has crosstalk with Ras2 [37], it will be of interest to investigate whether the retrograde signaling links RAS signaling and Yno1.

Our findings suggest that a severe defect in mitochondrial respiration impairs the maintenance of RLS by the accumulation of intracellular ROS rather than the loss of rDNA silencing. In addition, we suggest that the NADPH oxidase Yno1 significantly contributes to replicative aging by regulating ROS production in cells lacking respiratory activity along with RAS signaling. This study highlights the complex and multifaceted effects of ROS accumulation in yeast, which are relevant to the physiology of aging of higher organisms. Given that dysfunctional mitochondria are known to be connected with age-related metabolic and degenerative diseases, our results also provide implications for the mechanisms underlying cancerogenesis, neurodegenerative disease, diabetes, and obesity.

## Supporting information

S1 Fig Comparative analysis of the mitochondrial respiration capacity in used strains. 10-fold serial dilutions of wild-type (WT), rho^0^, *cyc3*Δ, *shy1*Δ, *cox5a*Δ, and *cyc1*Δ cells were spotted on YPG medium. The respiratory capacity of the indicated strains was assessed by monitoring the growth of 10-fold serial dilution of cells on YPG media. YPD medium was used as a control.
(PDF)

S2 Fig Determination of intracellular ROS level by flow cytometry. (A and B) Wild-type (WT) cells were grown and their fluorescence was analyzed without the indicated ROS probes. Based on this, the fluorescence output was set to zero. Any cells that have a value above zero were counted as P2. (C) Intracellular ROS levels in the indicated strains were detected with DHE. (D) Cells with high ROS were calculated as a percentage of cells with higher fluorescence intensity than the maximum fluorescence intensity of control sample without the ROS indicator. Values represent the average of three independent experiments, and error bars indicate the standard deviation. All asterisks indicate *P*<0.01, compared with WT cells (one-way ANOVA).
(PDF)

S3 Fig Quantification of protein oxidation, TOR, and PKA activity. (A) Carbonylated proteins in wild-type (WT), rho^0^, *cyc3*Δ, *shy1*Δ, *cox5a*Δ, *cyc1*Δ, and WT cells treated with 3 μg/ml AA or 10 μg/ml OM were detected using Oxidized Protein Detection kit. The relative change in protein oxidation was calculated as the ratio of carbonylated proteins in the indicated strain to those of WT cells. (B) Total protein was extracted from WT, rho^0^, *cyc3*Δ, *shy1*Δ, WT cells treated with 3 μg/ml AA or 10 μg/ml OM, and WT cells under nitrogen starvation. All cells harbor pRS416-SCH9^T570A^-5HA. Immunoblotting was performed using a mouse anti-HA antibody. Then the relative ratio of phosphorylated to unphosphorylated forms of Sch9 was calculated. (C) Total protein was extracted from WT, rho^0^, *cyc3*Δ, *shy1*Δ, WT cells treated with 3 μg/ml AA or 10 μg/ml OM, WT cells expressing constitutively active *RAS2*^*val19*^ (19V), and WT cells under glucose starvation. All cells harbor pRS423-CUP1-6xMYC-cki1^2-200(S125/130A)^. Immunoblotting was performed using a mouse anti-Myc antibody. Then the relative ratio of phosphorylated to unphosphorylated forms of Cki1 was calculated. All values represent the average of three independent experiments, and error bars indicate the standard deviation. All asterisks indicate *P*<0.01, compared with WT cells (one-way ANOVA).
(PDF)

S4 Fig Determination of intracellular ROS levels affected by RAS and Yno1 in rho^0^ cells using H_2_DCFDA. (A, B, and C) Intracellular ROS levels in the indicated strains were detected with H_2_DCFDA. Fluorescence was analyzed using a BD FACS Canto II flow cytometer.
(PDF)

S5 Fig Determination of intracellular ROS levels affected by RAS and Yno1 in rho^0^ cells using DHE. (A) Intracellular ROS levels in the indicated strains were detected with DHE. Fluorescence was analyzed using a BD FACS Canto II flow cytometer. (B) Cells with high ROS were calculated as a percentage of cells with higher fluorescence intensity than the maximum fluorescence intensity of control sample without the ROS indicator. Values represent the average of three independent experiments, and error bars indicate the standard deviation. All asterisks indicate *P*<0.01, compared with rho^0^ cells (one-way ANOVA).
(PDF)

S6 Fig Quantification of protein oxidation affected by RAS and Yno1 in rho^0^ cells. (A and B) Carbonylated proteins in the indicated strains were detected using Oxidized Protein Detection kit. Then the relative change in protein oxidation was calculated as the ratio of carbonylated proteins in the indicated strain to those of WT cells. Values represent the average of three independent experiments, and error bars indicate the standard deviation. All asterisks indicate *P*<0.01, compared with rho^0^ cells (one-way ANOVA).
(PDF)

S1 Table Yeast strains used in this study. (DOCX)

S2 Table Primers used for plasmid construction. (DOCX)

S3 Table Primers used for quantitative PCR. (DOCX)
