# Supplementary figures and images for: Mitochondrial dysfunction reduces yeast replicative lifespan by elevating RAS-dependent ROS production by the ER-localized NADPH oxidase Yno1
Source: PLoS One. 2018 Jun 18;13(6):e0198619. doi: 10.1371/journal.pone.0198619 (PMC6005541; doi:10.1371/journal.pone.0198619)

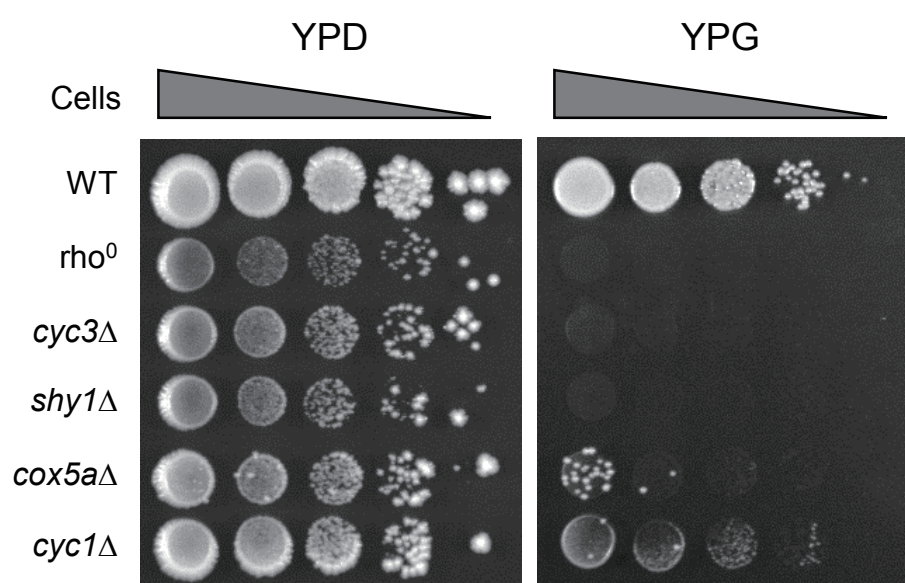

S1 Fig.

Supplement: S1 Fig — 10-fold serial dilutions of wild-type (WT), rho0, cyc3Δ, shy1Δ, cox5aΔ, and cyc1Δ cells were spotted on YPG medium. The respiratory capacity of the indicated strains was assessed by monitoring the growth of 10-fold serial dilution of cells on YPG media. YPD medium was used as a control. (PDF) [file pone.0198619.s001.pdf]

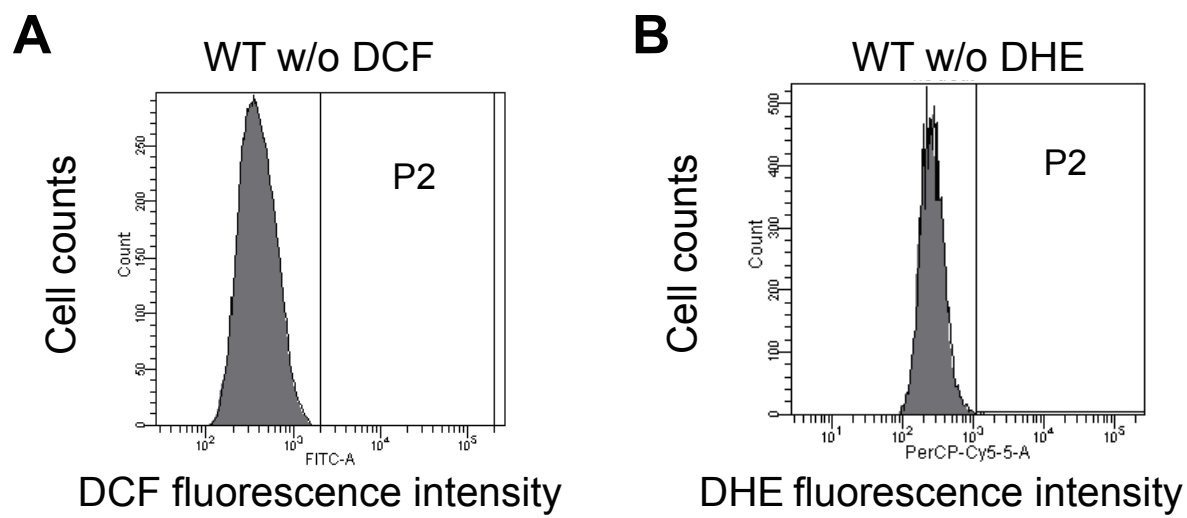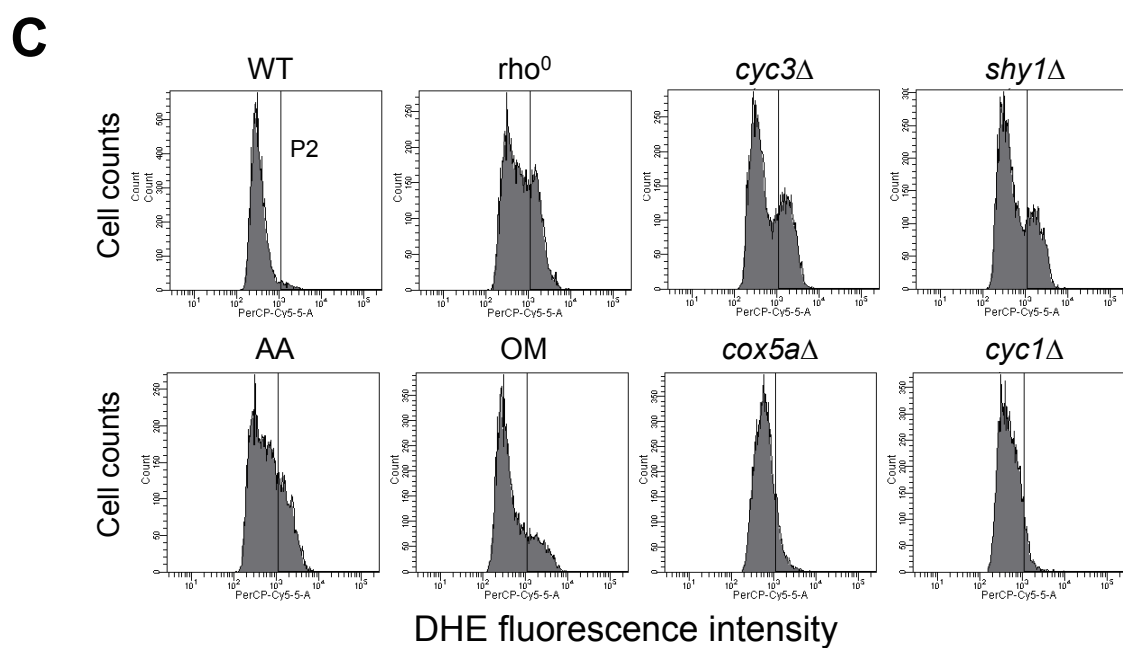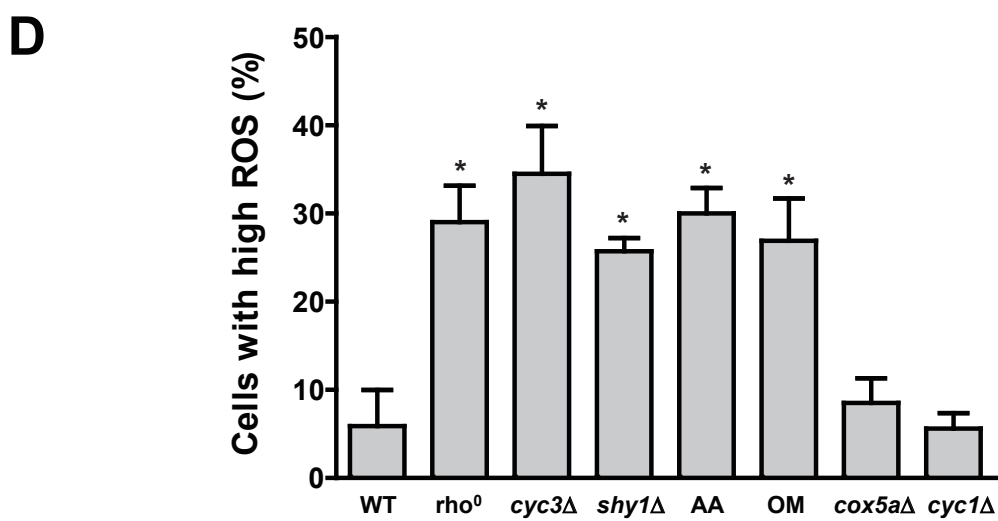

S2 Fig.

Supplement: S2 Fig — (A and B) Wild-type (WT) cells were grown and their fluorescence was analyzed without the indicated ROS probes. Based on this, the fluorescence output was set to zero. Any cells that have a value above zero were counted as P2. (C) Intracellular ROS levels in the indicated strains were detected with DHE. (D) Cells with high ROS were calculated as a percentage of cells with higher fluorescence intensity than the maximum fluorescence intensity of control sample without the ROS indicator. Values represent the average of three independent experiments, and error bars indicate the standard deviation. All asterisks indicate P<0.01, compared with WT cells (one-way ANOVA). (PDF) [file pone.0198619.s002.pdf]

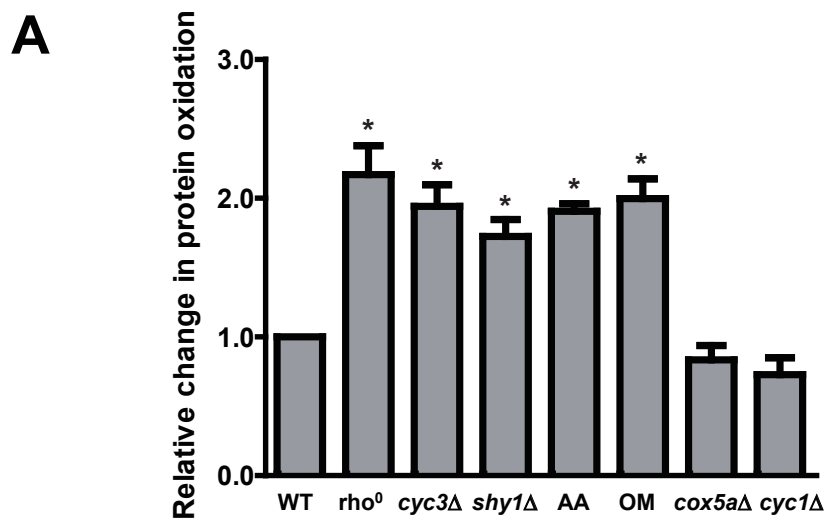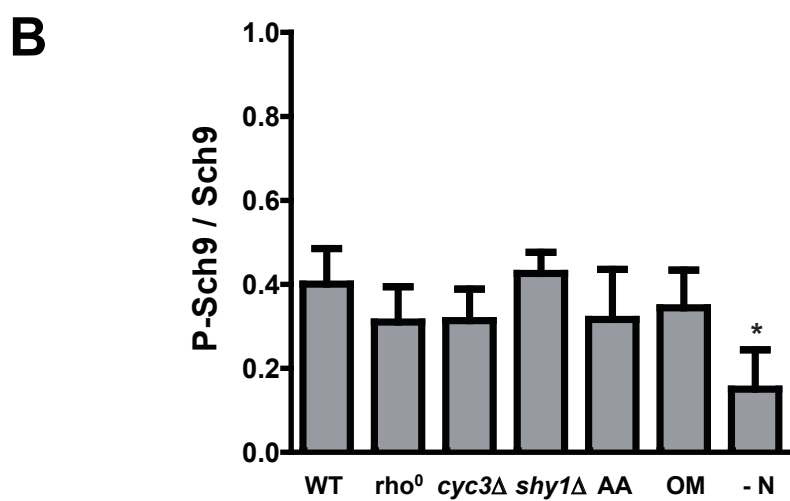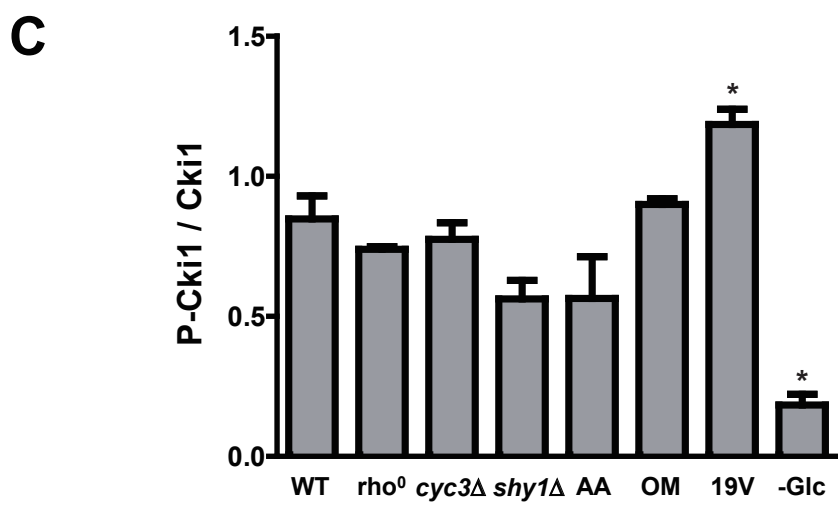

S3 Fig.

Supplement: S3 Fig — (A) Carbonylated proteins in wild-type (WT), rho0, cyc3Δ, shy1Δ, cox5aΔ, cyc1Δ, and WT cells treated with 3 μg/ml AA or 10 μg/ml OM were detected using Oxidized Protein Detection kit. The relative change in protein oxidation was calculated as the ratio of carbonylated proteins in the indicated strain to those of WT cells. (B) Total protein was extracted from WT, rho0, cyc3Δ, shy1Δ, WT cells treated with 3 μg/ml AA or 10 μg/ml OM, and WT cells under nitrogen starvation. All cells harbor pRS416-SCH9T570A-5HA. Immunoblotting was performed using a mouse anti-HA antibody. Then the relative ratio of phosphorylated to unphosphorylated forms of Sch9 was calculated. (C) Total protein was extracted from WT, rho0, cyc3Δ, shy1Δ, WT cells treated with 3 μg/ml AA or 10 μg/ml OM, WT cells expressing constitutively active RAS2val19 (19V), and WT cells under glucose starvation. All cells harbor pRS423-CUP1-6xMYC-cki12-200(S125/130A). Immunoblotting was performed using a mouse anti-Myc antibody. Then the relative ratio of phosphorylated to unphosphorylated forms of Cki1 was calculated. All values represent the average of three independent experiments, and error bars indicate the standard deviation. All asterisks indicate P<0.01, compared with WT cells (one-way ANOVA). (PDF) [file pone.0198619.s003.pdf]

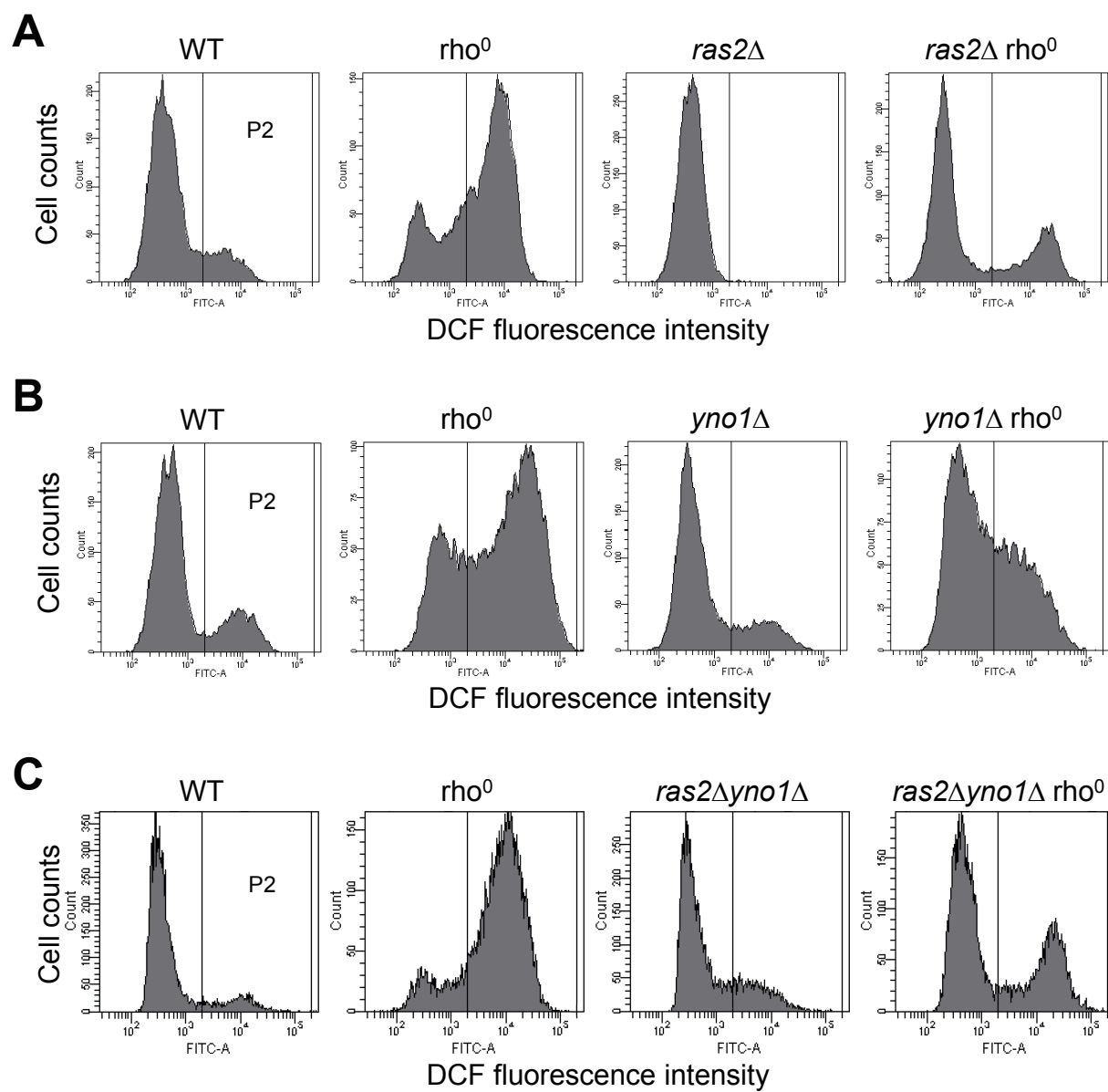

S4 Fig.

Supplement: S4 Fig — (A, B, and C) Intracellular ROS levels in the indicated strains were detected with H2DCFDA. Fluorescence was analyzed using a BD FACS Canto II flow cytometer. (PDF) [file pone.0198619.s004.pdf]

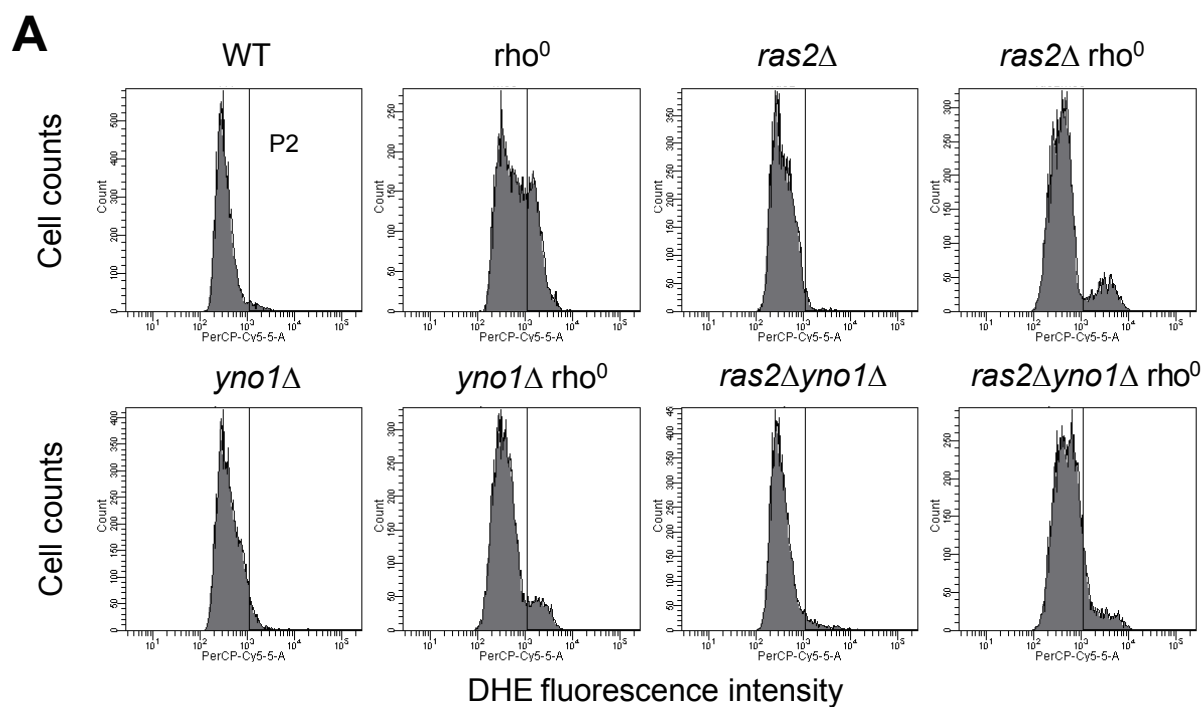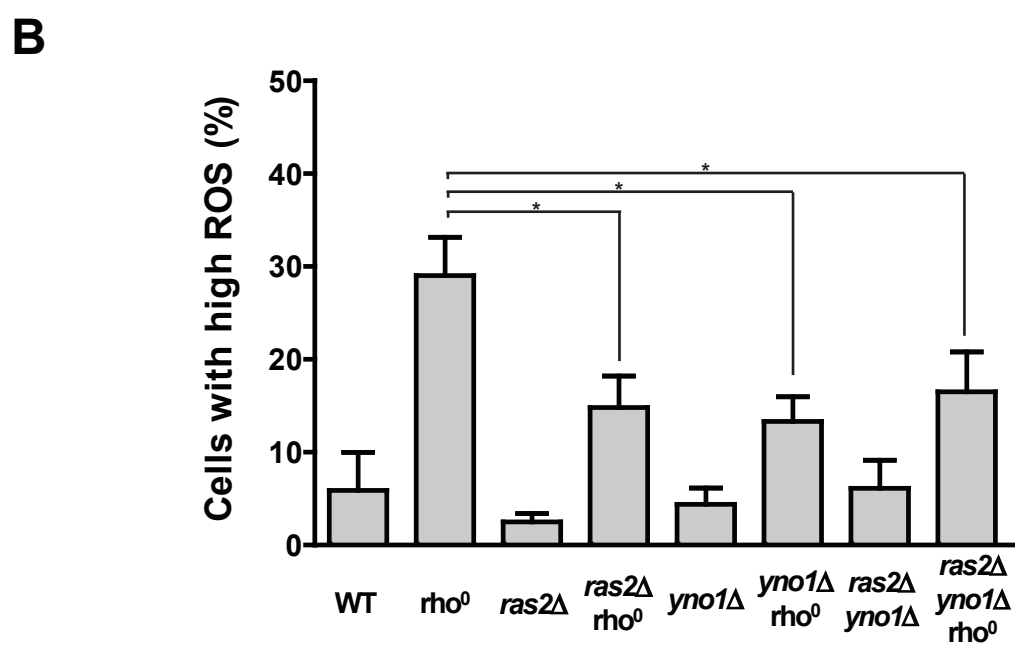

S5 Fig.

Supplement: S5 Fig — (A) Intracellular ROS levels in the indicated strains were detected with DHE. Fluorescence was analyzed using a BD FACS Canto II flow cytometer. (B) Cells with high ROS were calculated as a percentage of cells with higher fluorescence intensity than the maximum fluorescence intensity of control sample without the ROS indicator. Values represent the average of three independent experiments, and error bars indicate the standard deviation. All asterisks indicate P<0.01, compared with rho0 cells (one-way ANOVA). (PDF) [file pone.0198619.s005.pdf]

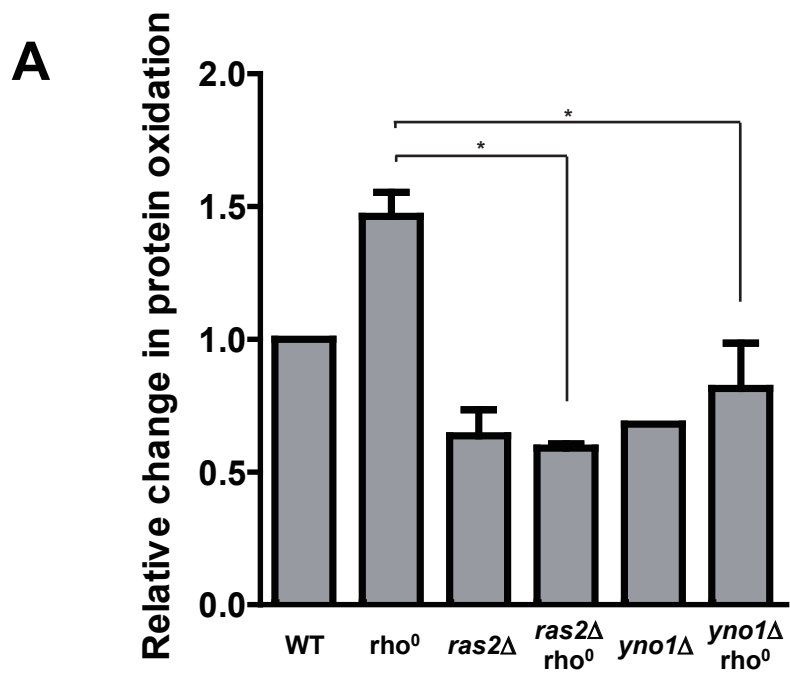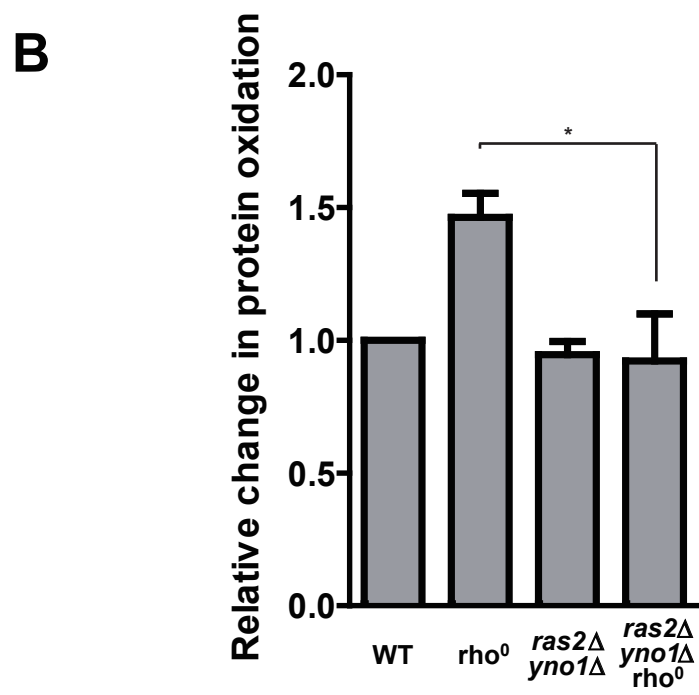

S6 Fig.

Supplement: S6 Fig — (A and B) Carbonylated proteins in the indicated strains were detected using Oxidized Protein Detection kit. Then the relative change in protein oxidation was calculated as the ratio of carbonylated proteins in the indicated strain to those of WT cells. Values represent the average of three independent experiments, and error bars indicate the standard deviation. All asterisks indicate P<0.01, compared with rho0 cells (one-way ANOVA). (PDF) [file pone.0198619.s006.pdf]
