# Supplementary material for: Mitochondrial dysfunction reduces yeast replicative lifespan by elevating RAS-dependent ROS production by the ER-localized NADPH oxidase Yno1
Source: PLoS One. 2018 Jun 18;13(6):e0198619. doi: 10.1371/journal.pone.0198619 (PMC6005541; doi:10.1371/journal.pone.0198619)
Supplement: S1 Table — (DOCX) [file pone.0198619.s007.docx]

**S1 Table. Yeast strains used in this study.**

| **Strain** | **Genotype** | **Source** |
| --- | --- | --- |
| BY4741 | *MAT***a** *his3*Δ*1 leu2*Δ*0 met15*Δ*0 ura3*Δ*0* | EUROSCARF |
| HY1680 | *MAT***a** *his3*Δ*1 leu2*Δ*0 met15*Δ*0 ura3*Δ*0 rho^0^* | This study |
| HY1681 | *MAT***a** *his3*Δ*1 leu2*Δ*0 met15*Δ*0 ura3*Δ*0 cyc3*Δ*::URA3* | This study |
| HY1682 | *MAT***a** *his3*Δ*1 leu2*Δ*0 met15*Δ*0 ura3*Δ*0 shy1*Δ*::URA3* | This study |
| HY1684 | *MAT***a** *his3*Δ*1 leu2*Δ*0 met15*Δ*0 ura3*Δ*0 cox5a*Δ*::URA3* | This study |
| HY1683 | *MAT***a** *his3*Δ*1 leu2*Δ*0 met15*Δ*0 ura3*Δ*0 cyc1*Δ*::URA3* | This study |
| DMY2798 | *MAT***a** *ade2-1 ura3-1 trp1-1 leu2-3,112 his3-11 can1-100 leu2::mURA3* | [1] |
| DMY2804 | *MAT***a** *ade2-1 ura3-1 trp1-1 leu2-3,112 his3-11 can1-100 RDN1-NTS1::mURA3* | [1] |
| DMY2800 | *MAT***a** *ade2-1 ura3-1 trp1-1 leu2-3,112 his3-11 can1-100 RDN1-NTS2::mURA3* | [1] |
| HY1685 | *MAT***a** *ade2-1 ura3-1 trp1-1 leu2-3,112 his3-11 can1-100 leu2::mURA3 rho^0^* | This study |
| HY1716 | *MAT***a** *ade2-1 ura3-1 trp1-1 leu2-3,112 his3-11 can1-100 RDN1-NTS1::mURA3 rho^0^* | This study |
| HY1717 | *MAT***a** *ade2-1 ura3-1 trp1-1 leu2-3,112 his3-11 can1-100 RDN1-NTS2::mURA3 rho^0^* | This study |
| HY1718 | *MAT***a** *ade2-1 ura3-1 trp1-1 leu2-3,112 his3-11 can1-100 leu2::mURA3 cyc3*Δ*::TRP1* | This study |
| HY1719 | *MAT***a** *ade2-1 ura3-1 trp1-1 leu2-3,112 his3-11 can1-100 RDN1-NTS1::mURA3 cyc3*Δ*::TRP1* | This study |
| HY1720 | *MAT***a** *ade2-1 ura3-1 trp1-1 leu2-3,112 his3-11 can1-100 RDN1-NTS2::mURA3 cyc3*Δ*::TRP1* | This study |
| HY1721 | *MAT***a** *ade2-1 ura3-1 trp1-1 leu2-3,112 his3-11 can1-100 leu2::mURA3 shy1*Δ*::TRP1* | This study |
| HY1722 | *MAT***a** *ade2-1 ura3-1 trp1-1 leu2-3,112 his3-11 can1-100 RDN1-NTS1::mURA3 shy1*Δ*::TRP1* | This study |
| HY1723 | *MAT***a** *ade2-1 ura3-1 trp1-1 leu2-3,112 his3-11 can1-100 RDN1-NTS2::mURA3 shy1*Δ*::TRP1* | This study |
| HY1727 | *MAT***a** *ade2-1 ura3-1 trp1-1 leu2-3,112 his3-11 can1-100 leu2::mURA3 cox5a*Δ*::TRP1* | This study |
| HY1728 | *MAT***a** *ade2-1 ura3-1 trp1-1 leu2-3,112 his3-11 can1-100 RDN1-NTS1::mURA3 cox5a*Δ*::TRP1* | This study |
| HY1729 | *MAT***a** *ade2-1 ura3-1 trp1-1 leu2-3,112 his3-11 can1-100 RDN1-NTS2::mURA3 cox5a*Δ*::TRP1* | This study |
| HY1724 | *MAT***a** *ade2-1 ura3-1 trp1-1 leu2-3,112 his3-11 can1-100 leu2::mURA3 cyc1*Δ*::TRP1* | This study |
| HY1725 | *MAT***a** *ade2-1 ura3-1 trp1-1 leu2-3,112 his3-11 can1-100 RDN1-NTS1::mURA3 cyc1*Δ*::TRP1* | This study |
| HY1726 | *MAT***a** *ade2-1 ura3-1 trp1-1 leu2-3,112 his3-11 can1-100 RDN1-NTS2::mURA3 cyc1*Δ*::TRP1* | This study |
| HY0245 | *MAT***a** *ade2-1 ura3-1 trp1-1 leu2-3,112 his3-11 can1-100 leu2::mURA3 sir2*Δ*::TRP1* | This study |
| HY0291 | *MAT***a** *ade2-1 ura3-1 trp1-1 leu2-3,112 his3-11 can1-100 RDN1-NTS1::mURA3 sir2*Δ*::TRP1* | This study |
| HY0247 | *MAT***a** *ade2-1 ura3-1 trp1-1 leu2-3,112 his3-11 can1-100 RDN1-NTS2::mURA3 sir2*Δ*::TRP1* | This study |
| DMY3010 | *MAT****a*** *ade2-1 ura3-1 trp1-1 leu2-3,112 his3-11 can1-100 RAD5+ with RDN1::ADE2* | [1] |
| HY1906 | *MAT****a*** *ade2-1 ura3-1 trp1-1 leu2-3,112 his3-11 can1-100 RAD5+ with RDN1::ADE2 rho^0^* | This study |
| HY1907 | *MAT****a*** *ade2-1 ura3-1 trp1-1 leu2-3,112 his3-11 can1-100 RAD5+ with RDN1::ADE2 cyc3*Δ*::URA3* | This study |
| HY1908 | *MAT****a*** *ade2-1 ura3-1 trp1-1 leu2-3,112 his3-11 can1-100 RAD5+ with RDN1::ADE2 shy1*Δ*::URA3* | This study |
| HY1909 | *MAT****a*** *ade2-1 ura3-1 trp1-1 leu2-3,112 his3-11 can1-100 RAD5+ with RDN1::ADE2 cox5a*Δ*::URA3* | This study |
| HY1910 | *MAT****a*** *ade2-1 ura3-1 trp1-1 leu2-3,112 his3-11 can1-100 RAD5+ with RDN1::ADE2 cyc1*Δ*::URA3* | This study |
| HY0236 | *MAT****a*** *ade2-1 ura3-1 trp1-1 leu2-3,112 his3-11 can1-100 RAD5+ with RDN1::ADE2 sir2*Δ*::TRP1* | This study |
| HY1730 | *MAT***a** *his3*Δ*1 leu2*Δ*0 met15*Δ*0 ura3*Δ*0 pRS416; SCH9^T570A^-5HA* | This study |
| HY1731 | *MAT***a** *his3*Δ*1 leu2*Δ*0 met15*Δ*0 ura3*Δ*0 rho^0^ pRS416; SCH9^T570A^-5HA* | This study |
| HY1732 | *MAT***a** *his3*Δ*1 leu2*Δ*0 met15*Δ*0 ura3*Δ*0 cyc3*Δ*::HIS3 pRS416; SCH9^T570A^-5HA* | This study |
| HY1911 | *MAT***a** *his3*Δ*1 leu2*Δ*0 met15*Δ*0 ura3*Δ*0 shy1*Δ*::HIS3 pRS416; SCH9^T570A^-5HA* | This study |
| HY1736 | *MAT***a** *his3*Δ*1 leu2*Δ*0 met15*Δ*0 ura3*Δ*0 pRS423-CUP1-6xMYC-cki1^2-200(S125/130A)^* | This study |
| HY1709 | *MAT***a** *his3*Δ*1 leu2*Δ*0 met15*Δ*0 ura3*Δ*0 rho^0^ pRS423-CUP1-6xMYC-cki1^2-200(S125/130A)^* | This study |
| HY1710 | *MAT***a** *his3*Δ*1 leu2*Δ*0 met15*Δ*0 ura3*Δ*0 cyc3*Δ*::URA3 pRS423-CUP1-6xMYC-cki1^2-200(S125/130A)^* | This study |
| HY1792 | *MAT***a** *his3*Δ*1 leu2*Δ*0 met15*Δ*0 ura3*Δ*0 shy1*Δ*::URA3 pRS423-CUP1-6xMYC-cki1^2-200(S125/130A)^* | This study |
| HY1739 | *MAT***a** *his3*Δ*1 leu2*Δ*0 met15*Δ*0 ura3*Δ*0 ras2*Δ*::URA3 pRS415GPD-RAS2^19V^ pRS423-CUP1-6xMYC-cki1^2-200(S125/130A)^* | This study |
| HY1158 | *MAT***a** *his3*Δ*1 leu2*Δ*0 met15*Δ*0 ura3*Δ*0 ras2*Δ*::URA3* | This study |
| HY1713 | *MAT***a** *his3*Δ*1 leu2*Δ*0 met15*Δ*0 ura3*Δ*0 ras2*Δ*::URA3 rho^0^* | This study |
| HY1711 | *MAT***a** *his3*Δ*1 leu2*Δ*0 met15*Δ*0 ura3*Δ*0 yno1*Δ*::URA3* | This study |
| HY1712 | *MAT***a** *his3*Δ*1 leu2*Δ*0 met15*Δ*0 ura3*Δ*0 yno1*Δ*::URA3 rho^0^* | This study |
| HY1904 | *MAT***a** *his3*Δ*1 leu2*Δ*0 met15*Δ*0 ura3*Δ*0 ras2*Δ*::HIS3 yno1*Δ*::URA3* | This study |
| HY1905 | *MAT***a** *his3*Δ*1 leu2*Δ*0 met15*Δ*0 ura3*Δ*0 ras2*Δ*::HIS3 yno1*Δ*::URA3 rho^0^* | This study |
|  |  |  |

1. Huang J, Brito IL, Villén J, Gygi SP, Amon A, Moazed D. Inhibition of homologous recombination by a cohesin-associated clamp complex recruited to the rDNA recombination enhancer. Genes Dev. 2006;20(20):2887-901.
