# Supplementary material for: Mitochondrial dysfunction reduces yeast replicative lifespan by elevating RAS-dependent ROS production by the ER-localized NADPH oxidase Yno1
Source: PLoS One. 2018 Jun 18;13(6):e0198619. doi: 10.1371/journal.pone.0198619 (PMC6005541; doi:10.1371/journal.pone.0198619)
Supplement: S2 Table — (DOCX) [file pone.0198619.s008.docx]

**S2 Table. Primers used for plasmid construction.**

| **Primer name** | **Sequence (5’→3’)^a^** |
| --- | --- |
| RAS2(G19V)-F | GTCGTTGGTGGTGTTGGTGTTGGTAAA |
| RAS2(G19V)-R | TTTACCAACACCAACACCACCAACGAC |
| RAS2+1200R(SalI) | GACTGTCGACGATGACTCTCTGCAATGTCC |
| RAS2-6(XbaI) | TCAGTCTAGATTTCTGTATATCTCCTTTCAATTC |
|  |  |

^a^ Sequences underlined represent the restriction enzyme sites for cloning of the PCR products.
