# Supplementary material for: Mitochondrial dysfunction reduces yeast replicative lifespan by elevating RAS-dependent ROS production by the ER-localized NADPH oxidase Yno1
Source: PLoS One. 2018 Jun 18;13(6):e0198619. doi: 10.1371/journal.pone.0198619 (PMC6005541; doi:10.1371/journal.pone.0198619)
Supplement: S3 Table — (DOCX) [file pone.0198619.s009.docx]

**S3 Table. Primers used for quantitative PCR.**

| **Primer** | **Sequence (5’→3’)** |
| --- | --- |
| **Primers for qPCR** |  |
| ACT1-CHK | TGACTGACTACTTGATGAAG |
| ACT1+1105R | ACAGAAGGATGGAACAAAGC |
| URA3+503 | CTGTTGACATTGCGAAGAGC |
| URA3+632R | TCTCCCTTGTCATCTAAACC |
